# Intelligence, &c.

**Published:** 1828-01-01

**Authors:** 


### INTELLIGENCE, &c.

### Dr. COOKE.

We have received a rather sharp remonstrance, amounting almost to a *reproof*, for a question which we asked the College of Physicians in our last number, namely, whether they could turn out a *more* erudite physician than Dr. Cooke, who never breathed the air of Oxford or Cambridge? It can hardly be supposed that we meant to hurt the feelings of Dr. Cooke by this allusion; and any thing we could now say by way of *retractation*, would probably make matters worse. In this dilemma we can only, like a true penitent, promise never so to offend again. We may be permitted, however, to remark that, as the question did not imply a *superiority*, but only an *equality* of erudition, on the part of Dr. Cooke, so we really imagine that the Doctor is too sensitive on this point, and that there is nothing in the passage which need render him uncomfortable as to his brother-fellows in the College. At all events, on our heads be the sin, and also the error—if we were in error.

### Mr. Guthrie's Action against the Lancet.

On the 18th December, after the lawyers had unfolded their briefs, at Guildhall, and all was clear for action, the proceedings were *suddenly* stopped, and the trial postponed till next term! The cause of this postponement is not, at present, (20th December) clearly ascertained, though rumours are afloat, to which we do not, in the present state of the business, consider ourselves bound to advert. We shall return to the subject in a short time.

### Literary Notices.

In the press, and speedily will be published, in royal octavo, with plates, Physiological Illustrations of the Organ of Hearing, more particularly of the Secretion of Cerumen, and its effects in rendering auditory perception accurate and acute, with further remarks on the Treatment of Diminution of Hearing, arising from imperfect Secretion, &c. with Cases: being a Sequel to the Guide, and to the Illustrations of Acoustic Surgery. By Thomas Buchanan, C.M.

In the press, and will be published in a few days, a Practical Treatise upon Stricture of the Rectum; illustrating, by Cases, the connexion of that Disease with Affections of the Urinary Organs, the Uterus, and with Piles. By Fred. Salmon, Surgeon to the General Dispensary, and formerly House-Surgeon to Saint Bartholomew's Hospital.

Dr. Armstrong is preparing for publication an octavo volume on the Remote Causes, Prevention, Nature, and Treatment of Diseases of the Stomach, Liver, and Bowels. This work, which will appear in the spring, will be preceded by a series of coloured drawings, in quarto, with copious letter-press, illustrative of the morbid anatomy of the stomach, liver, and bowels. They will be published in six monthly fasciculi, each containing about five plates accurately coloured from Nature: the first number will be ready early in December.

Mr. Grainger, Lecturer on Anatomy and Physiology, is preparing for publication the Elements of General Anatomy, in 1 vol. 8vo.

Mr. Dermott, Lecturer on Anatomy, has nearly ready the 4th number of his Anatomical Plates, comprising the Anatomy of the Abdominal Muscles, and the Parts immediately connected with Inguinal Hernia.

N. B. Books transmitted for review will be registered, as usual; but no literary notices will be inserted in the Fasciculi, except as advertisements on the cover. The Journal will be kept free from all matters not strictly connected with the Review and Periscope departments.

